# Impact of Early Incorporation of Nutrition Interventions as a Component of Cancer Therapy in Adults: A Review

**DOI:** 10.3390/nu12113403

**Published:** 2020-11-05

**Authors:** Julie Richards, Mary Beth Arensberg, Sara Thomas, Kirk W. Kerr, Refaat Hegazi, Michael Bastasch

**Affiliations:** 1 Abbott Nutrition, Bob Evans Farms, Columbus, OH 43212, USA; 2 Abbott Nutrition Division of Abbott, Columbus, OH 43219, USA; mary.arensberg@abbott.com (M.B.A.); sara.thomas@abbott.com (S.T.); kirk.kerr@abbott.com (K.W.K.); refaat.hegazi@abbott.com (R.H.); 3 Department of Medicine and Division of Radiation Oncology, University of Texas East Health, Athens, TX 75751, USA; bastaschmd@hotmail.com

**Keywords:** malnutrition, oncology, cancer care, nutrition interventions, early intervention, nutrition counseling, oral nutrition supplements, health outcomes

## Abstract

Malnutrition is prevalent among oncology patients and can adversely affect clinical outcomes, prognosis, quality of life, and survival. This review evaluates current trends in the literature and reported evidence around the timing and impact of specific nutrition interventions in oncology patients undergoing active cancer treatment. Previous research studies (published 1 January 2010–1 April 2020) were identified and selected using predefined search strategy and selection criteria. In total, 15 articles met inclusion criteria and 12/15 articles provided an early nutrition intervention. Identified studies examined the impacts of nutrition interventions (nutrition counseling, oral nutrition supplements, or combination of both) on a variety of cancer diagnoses. Nutrition interventions were found to improve body weight and body mass index, nutrition status, protein and energy intake, quality of life, and response to cancer treatments. However, the impacts of nutrition interventions on body composition, functional status, complications, unplanned hospital readmissions, and mortality and survival were inconclusive, mainly due to the limited number of studies evaluating these outcomes. Early nutrition interventions were found to improve health and nutrition outcomes in oncology patients. Future research is needed to further evaluate the impacts of early nutrition interventions on patients’ outcomes and explore the optimal duration and timing of nutrition interventions.

## 1. Introduction

Population aging and growth are driving the global burden of cancer, which is estimated to increase by more than 60% by 2040 and become the leading barrier to increasing life expectancy in this century [1]. At the same time, malnutrition persists as a growing crisis across the continuum of care. In the oncology population, the prevalence of malnourished patients or those at risk of malnutrition ranges from 25 to 70% [2,3,4,5,6,7,8,9]. Many patients with cancer are malnourished on diagnosis. Over the course of cancer and its treatment, malnutrition can also develop, continue, or worsen. However, malnutrition, particularly protein energy deficits and muscle loss, frequently remains underdiagnosed and undertreated among oncology patients [10]. This is a significant problem because malnutrition is related to multiple poor outcomes in patients with cancer. Indeed, malnutrition results in increased mortality rates, and 10–20% of deaths in cancer patients can be attributed to malnutrition vs. the malignancy itself [5,11,12].

Oncology patients can have complex nutrition problems, which often vary depending on the location and stage of the cancer. The side effects from cancer treatments can further exacerbate nutrition problems, including cancer cachexia. Cancer cachexia is characterized by a negative protein and energy intake combined with systemic inflammation and hyper-metabolism [13].

Thus, implementing nutrition interventions early is particularly critical for patients with cancer. Clinical guidelines, including those from the Academy of Nutrition and Dietetics (Academy) [14], the American Society for Parenteral and Enteral Nutrition (ASPEN) [15], and the European Society for Clinical Nutrition and Metabolism (ESPEN) [16], advocate for the importance of early nutrition screening and intervention in oncology patient populations.

Multiple nutrition interventions, including dietary counseling or advice, oral nutritional supplements (ONS), and enteral nutrition, have shown positive outcomes in malnourished hospitalized and community-dwelling adults with cancer [17,18,19]. Furthermore, systematic reviews have underscored the strength of evidence of such nutrition interventions on nutrition and health outcomes for oncology patients [20,21,22,23,24]. In addition to the positive effects of nutrition interventions on malnutrition, nutrition interventions have also been documented to improve outcomes such as quality of life (QoL) [18] and possibly survival [19] in cancer patients. However, it seems no previous review has evaluated the impact of early incorporation of nutrition interventions as a component of cancer therapy. The aim of this review was to evaluate the current evidence around the timing and impact of specific nutrition interventions in oncology patients undergoing active cancer treatment and to identify trends in the literature.

## 2. Materials and Methods

This study performed a basic review of the recent literature on specific nutrition interventions for oncology patients.

### 2.1. Search Strategy

A comprehensive electronic literature search was completed in April 2020 with a predefined search strategy. The databases that were searched included Allied and Complementary Medicine™ (EBSCO Information Services, Ipswich, MA, USA), BIOSIS Previews^®^ (EBSCO Information Services, Ipswich, MA, USA), Embase^®^ (Elsevier, Amsterdam, The Netherlands), EMCare^®^ (Ovid, New York City, NY, USA), International Pharmaceutical Abstracts (EBSCO Information Services, Ipswich, MA, USA), MEDLINE^®^ (Medical Literature Analysis and Retrieval System Online; National Library of Medicine, Bethesda, MD, USA), and ToxFile^®^ (ProQuest Dialog, Ann Arbor, MI, USA). Key search terms are listed in Table 1, and the full electronic search strategy is included in Appendix A. Search results were limited to research in the adult population published from developed countries between 1 January 2010 and 1 April 2020 and written in the English language. This specific date range was chosen because past reviews [20,21,22,23,24] have already evaluated nutrition interventions from studies dated previously and because early nutrition intervention specifically is a more modern practice due to evidence-based position papers and clinical guidelines [14,15,16,25,26,27] that have raised awareness and provided guidance for nutrition interventions. Manual searches were also performed on existing systematic reviews and studies recommended for consideration from clinical nutrition experts.

The review focused on studies that investigated health and nutrition outcomes of specified nutrition interventions in adults diagnosed with any type of cancer who were receiving or planned to receive active treatment (other than surgery alone) for their cancer. Additionally, timing of nutrition intervention was of interest. Early intervention was defined as a specified nutrition intervention initiated within the first week of cancer treatment or before, while late intervention was identified as a specified nutrition intervention provided after the first week of cancer treatment. This cutoff for early nutrition intervention was chosen based on previous research, whereby early nutrition intervention was typically defined as being at the start of therapy or before [28,29]. Studies providing either early or late nutrition interventions were included in this review to compare outcomes. Studies evaluating nutrition intervention alone were included and studies with nutrition intervention along with other separate interventions (such as vitamin or mineral supplementation, exercise or physical activity, behavioral or mental health interventions, or alternative medicine) were excluded, since the focus of this review was on more general nutrition interventions vs. multicomponent therapies.

Health and nutrition outcomes of interest were anthropometric measures, nutritional and functional status, protein or energy intake, muscle strength, quality of life (QoL) measures, hospital readmissions or unplanned hospitalizations, response to treatment, emergency department (ED) visits, complications or morbidity, mortality, and healthcare costs. Mortality was generally defined as overall survival, rather than progression-free survival.

### 2.2. Inclusion and Exclusion Criteria

Studies identified through the electronic and manual searches were compared against the predetermined eligibility criteria, which were aligned with the population, intervention, comparison, outcome, time (PICOT) model, and are summarized in Table 2.

### 2.3. Data Extraction

Four reviewers (J.R., M.B.A., S.T., and K.W.K.) independently screened abstracts and assessed full-text articles for eligibility. Data were extracted per the PICOT framework and documented in Table 3. Reviewers met after the screening and full-text assessment steps to discuss all studies and reach an agreement on studies for final inclusion.

## 3. Results

### 3.1. Literature Search

**Figure 1:**
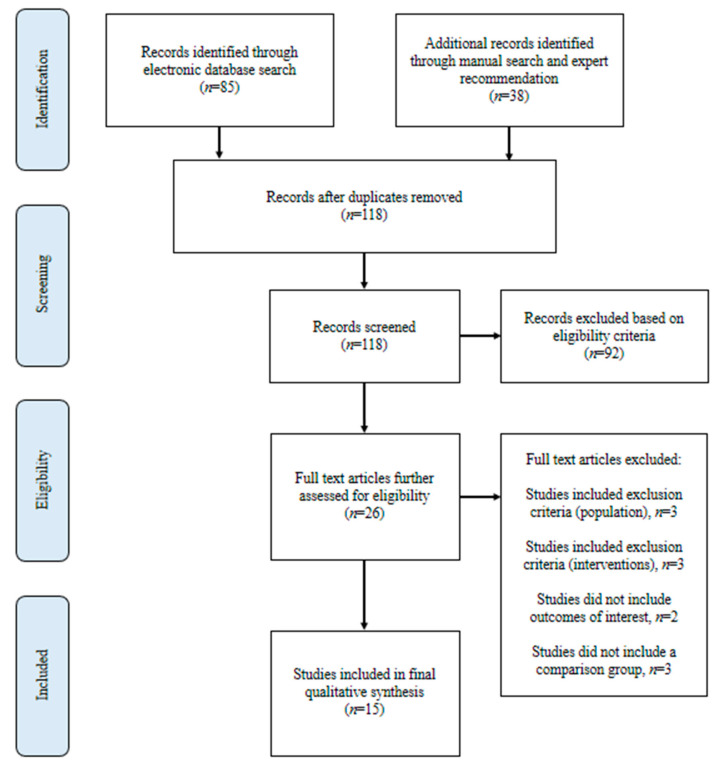
Flow diagram showing selection of studies for this review of early incorporation of nutrition interventions as a component of cancer therapy.

The literature search and selection steps are outlined in Figure 1. The electronic literature search resulted in 85 articles. In addition, 38 studies were recommended for consideration from clinical nutrition experts and manual search results, resulting in a total of 123 articles for consideration. After removal of duplicate publications, 118 articles were available for assessment. Only 15 studies met the predetermined eligibility criteria and were, thus, included in the final qualitative analysis.

### 3.2. Study Characteristics

Specific characteristics of the 15 eligible studies [28,29,30,31,32,33,34,35,36,37,38,39,40,41,42] meeting the inclusion criteria, which included publication in the last 10 years, are detailed in Table 3. The key provided at the bottom of Table 3 provides further information on statistical significance and nutrition intervention group comparisons. Among the 15 included studies, 10 were randomized controlled trials (RCTs) [30,31,32,33,34,35,36,38,39,40,42]. Sample sizes ranged from 13 to 341 participants. Cancer diagnoses included from head and neck cancers (HNC) [28,29,31,35,42], non-small cell lung cancer [36,39,40,41], gastrointestinal (GI) cancers [33,34,37,38], gynecologic cancer [33], pancreatic or bile duct cancers [32], and lymphoma or carcinoma [30].

For over half (9/15) of the studies, nutrition counseling was a nutrition intervention strategy [29,30,31,32,33,34,38,41,42]. ONS was used in all 15 studies [28,29,30,31,32,33,34,35,36,37,38,39,40,41,42], although some studies (5/15) only provided ONS if identified as needed or desired [29,30,33,41,42]. Eight studies (8/15) used ONS containing eicosapentaenoic acid (ONS-EPA) [33,35,36,37,38,39,40,41]. Some of these studies compared counseling + ONS-EPA to standard care [33,41], or to nutrition counseling only [38], while others compared ONS-EPA to no nutrition treatment [10], an isocaloric diet [36], or an isocaloric ONS [35,39,40]. Only one study specifically compared the results of early vs. late nutrition intervention [28].

## 4. Outcomes

### 4.1. Anthropometrics, Body Composition, and Nutritional Status

Fourteen studies (14/15) evaluated the effects of nutrition interventions on anthropometric measures, including body weight, BMI, and body composition [28,29,30,31,32,33,34,35,36,37,38,39,41,42].

Seven (7/15) studies reported reduced weight loss in the intervention group, and these interventions included early counseling combined with ONS [29,31,33,42], providing ONS at the beginning of treatment [28], or providing ONS-EPA [36,39]. Tanaka et al. [41] and Trabal et al. [38] found that a nutrition prescription of ONS-EPA resulted in weight gain. Four studies reported on changes in BMI, with two studies finding an increase in BMI as a result of the nutrition interventions [34,41] and two finding a reduction in BMI decline after the nutrition intervention [28,35].

Five (5/15) studies evaluated the effects of nutrition interventions on body composition [32,33,36,37,39]. Sanchez-Lara et al. [36] and Shirai et al. [37] found significant increases in lean body mass (LBM) after providing nutrition interventions that included ONS-EPA compared to control groups, while van der Meij et al. [39] found significantly less loss of fat free mass (FFM) in the intervention group receiving ONS-EPA. However, Kim et al. and Poulsen et al. did not find a difference in FFM after providing the nutrition interventions, but Kim et al. did observe a significant increase in fat mass [32,33].

Three studies (3/15) evaluated nutritional status [32,34,42]. Two studies used the patient-generated subjective global assessment (PG-SGA) method and found significant improvements in nutritional status in the nutrition intervention groups, which received nutrition counseling with ONS [32] and nutrition counseling plus education [34]. Additionally, van der Berg et al. defined malnutrition as unintended weight loss > 5% within 1 month and found a significant difference in malnutrition two weeks after the treatment between the nutrition intervention group receiving counseling plus ONS vs. the control group [42].

### 4.2. Nutritional Intake

Eight studies (8/15) evaluated energy intake [30,31,32,33,34,36,38,39], with six studies finding a significant increase in energy intake in the nutrition intervention groups [30,31,32,33,34,36] and one study finding an increased energy intake that was not significant [38]. Seven studies (7/15) evaluated protein intake [31,32,33,34,36,38,39], with five studies finding a significant increase in protein intake in the nutrition intervention groups [31,32,33,34,36] and one study finding an increased protein intake that was not significant [38]. Further, van der Meij et al. did not find a difference in energy or protein intake between groups; however, this study was comparing interventions of an ONS-EPA vs. an isocaloric ONS [39].

### 4.3. Functional Status

Three studies (3/15) evaluated functional status through physical activity, physical function, or handgrip strength [31,40,41]. Of these, van der Meij et al. found a significant increase in physical activity and physical function in the group provided ONS-EPA compared to controls provided with an isocaloric ONS [40]. Three studies used handgrip strength as a measure of functional status; Cereda et al. [31] saw an increase in handgrip strength after the nutrition intervention, while Tanaka et al. [41] saw a significant decrease and van der Meij et al. [40] did not find a difference.

### 4.4. QoL

Eight studies (8/15) evaluated QoL [31,32,33,34,36,38,40,41], with three studies reporting an overall improvement in QoL in the nutrition intervention groups compared to the control groups [31,34,41]. However, Poulsen et al. did not find a difference in QoL between the intervention and control groups [33]. 

Four studies (4/15) reported improvements in subscales of the QoL questionnaire [32,36,38,40]. Three studies reported improved fatigue in the nutrition intervention groups compared to control groups [32,36,38]. Two studies reported better physical and social function results in the intervention groups vs. the controls groups [38,40]. Improvements in appetite [36], global health status [36,40], and cognitive function [40] were also reported as a result of nutrition interventions. However, Trabel et al. found that loss of appetite worsened in the nutrition intervention group compared to the control group [38].

### 4.5. Response to Cancer Treatment

Ten studies (10/15) evaluated response to cancer treatment, including treatment tolerance or breaks or delays in treatment [28,29,30,31,34,36,37,38,40,41]. Five studies found a statistically significant improvement in treatment tolerance for those in the nutrition intervention groups vs. the control groups [28,29,34,36,40] and two other studies reported an improvement in treatment tolerance that was not significant [31,38]. Shirai et al. found an improved treatment tolerance in subjects who received ONS-EPA and had a modified Glasgow prognostic score (mGPS) of 1 or 2 compared to controls [37]. However, Tanaka et al. found no difference in treatment tolerance between the nutrition intervention group receiving counseling and ONS-EPA (as needed) and the control group receiving standard care [41].

Three studies reported on treatment breaks or delays [28,29,40]. Meng et al. found significantly fewer treatment delays for toxicity and significantly fewer CRT breaks (>3 days) in the early nutrition intervention group vs. the late nutrition intervention group [28], while Paccagnella et al. found significantly fewer treatment delays in the nutrition intervention group receiving counseling + ONS vs. the control group receiving standard care [29]. However, van der Meij et al. found no difference in treatment delays between the nutrition intervention group receiving ONS-EPA and the control group receiving isocaloric ONS [40].

Two studies reported on the results of cancer treatments. Bourdel-Marchasson et al. [30] did not observe a difference in full remission at the end of treatment between the nutrition intervention vs. control group, and Sanchez-Lara et al. [36] similarly did not observe a difference in overall tumor response rates between groups.

### 4.6. Complications and Unplanned Hospitalizations

One study (1/15) evaluated complications and found that the nutrition intervention group receiving counseling + ONS had significantly lower infections compared to the control group [30]. Three studies (3/15) evaluated unplanned hospitalizations [28,29,40]. Meng et al. [28] and Paccagnella et al. [29] found a statistically significant reduction in unplanned hospitalizations between nutrition intervention groups vs. control groups; however, van der Meij et al. [40] did not find a difference.

### 4.7. Mortality and Survival

Four studies (4/15) evaluated mortality and survival [30,34,36,37]. Ravasco et al. evaluated individualized nutrition counseling (group 1) vs. ONS with usual diet (group 2) vs. usual diet only (group 3) and found that the disease-specific survival time in group 3 < group 2 < group 1 (*p* < 0.05) [34]. However, three studies found no difference in mortality and survival between nutrition intervention groups and control groups [30,36,37].

When Sanchez-Lara et al. compared patients who consumed the complete dose of ONS-EPA (2 containers/day) vs. the control group, a trend was observed toward an increase in progression-free survival (*p* = 0.07) [36]. Additionally, Shirai et al. found a significantly better prognosis in subjects who received ONS-EPA and had a mGPS of 1 or 2 compared to controls [37].

### 4.8. Timing of Nutrition Intervention

The majority of the studies (12/15) provided an early nutrition intervention to the intervention groups [28,29,30,31,33,34,36,38,39,40,41,42]. Meng et al. specifically evaluated the impact of early vs. late nutrition interventions and found favorable outcomes for the early intervention group, including significantly reduced weight loss, significantly improved treatment tolerance, and significantly fewer unplanned hospitalizations [28]. Kim et al. provided early nutrition intervention to about 60% of subjects, since some subjects enrolled in the study were on later cycles of chemotherapy (CT) [32]. This study compared counseling plus ONS vs. counseling only. Since the different timing could have affected the results of the study, Kim et al. subdivided the subjects according to their cycle of CT. Body weight, skeletal muscle mass, and fat mass were increased significantly among subjects in their first cycle of CT who were receiving ONS, while only fat mass was increased among subjects in their second cycle or higher who were receiving ONS [32].

Roca-Rodriguez et al. evaluated ONS-EPA vs. standard ONS and provided these interventions 14 days after the start of RT, which in our review is categorized as a late nutrition intervention. Roca-Rodriguez reported a smaller decline in BMI for the ONS-EPA group vs. the standard ONS group, although this difference was not significant [35]. Shirai et al. did not specifically report when the nutrition intervention was initiated, so it is unclear if the intervention in their study was provided early or late [37].

## 5. Discussion

This review found that nutrition interventions had a positive impact on anthropometrics (body weight and BMI), nutrition status, protein and energy intake, QoL, and response to cancer treatments (treatment tolerance and treatment breaks/delays). The interventions used were ONS or ONS-EPA, nutrition counseling, or a combination of counseling and ONS. These results highlight the importance of early incorporation of nutrition interventions as a component of cancer therapy for the oncology patient population.

Inconclusive results were reported regarding body composition, functional status, complications, unplanned hospital readmissions, and mortality or survival. It is important to note that only a few studies focused on these measures and that these studies included small- to medium-sized samples. Additionally, the inconsistent and highly variable nutrition interventions and follow-up periods should be taken into consideration. Before conclusions can be drawn about the effects of nutrition interventions on these outcomes, further studies are necessary with larger sample sizes, consistent nutrition interventions of sufficient duration, and consistent timing of nutrition intervention and follow-up. Other outcomes of interest included in Table 2 but not addressed in the outcomes section were not reported in any of the identified studies.

Overall, the results of this review build upon the current body of evidence suggesting that nutrition interventions can result in improved outcomes for oncology patients. Other reviews of nutrition interventions for oncology patients have also shown positive results [21,22]. Due to the variations between study designs and interventions used in the studies we included, it is difficult to identify which nutrition intervention(s) led to the most beneficial outcomes. In the one long-term follow-up study included in our review by Ravasco et al. comparing individualized nutrition counseling and education (group 1), ONS plus usual diet (group 2), and usual diet only (group 3), it was found that group 1 had a significantly higher nutrition status, BMI, energy and protein intake, treatment tolerance, and QoL than group 2 and group 3 at the time of follow-up (4.9–8.2 years) [34]. Additionally, group 1 had significantly lower mortality [34]. These results support the long-term benefits of nutrition counseling for oncology patients and reinforce the Academy’s Oncology Evidence-Based Nutrition Practice Guideline for Adults [14], which recommends oncology patients undergoing CT or radiation treatment should receive medical nutrition therapy (MNT) from Registered Dietitian Nutritionists (RDNs) based on strong, conditional evidence. As identified in a number of studies included in our review, ONS also helps to increase calorie and protein intake and can be particularly beneficial for patients before and while actively receiving cancer treatments who need more nutrition, have a loss of appetite, or are experiencing other treatment-related side effects. Moreover, to improve outcomes in oncology patients, dietary counseling that includes the use of ONS should be a first step toward increased energy and protein intake [14,16]. Indeed, many of the studies included in our review used counseling combined with ONS as the nutrition intervention [29,30,31,32,33,38,41,42].

Eight studies included in our review used ONS-EPA. Eicosapentaenoic acid (EPA) is a polyunsaturated long-chain omega-3 fatty acid. Its use has gained momentum in the oncology patient population due to its anti-inflammatory properties and evidence that it can prevent muscle loss [43], making EPA of particular interest for preventing and treating cancer cachexia. Previous systematic reviews have concluded there is insufficient evidence to support a recommendation that long-chain omega-3 fatty acids can treat cancer cachexia; however, these previous reviews did not include any studies published after June 2010 [44,45,46]. Nonetheless, evidence supports other benefits of long-chain omega-3 fatty acid use for oncology patients. Systematic reviews on the supplementation of long-chain omega-3 fatty acids in oncology patients found improved body weight, post-surgical morbidity, and QoL [47] and preserved body composition [48] as a result of supplementation. Similarly, the Academy’s Oncology Evidence-Based Nutrition Practice Guideline for Adults [14] recommends, based on strong empirical evidence, the use of commercial supplements with EPA for patients with adequate dietary intake who are still experiencing weight or lean body mass loss. ESPEN’s Guidelines on Nutrition in Cancer Patients [16] also recommend supplementation with long-chain N-3 fatty acids to stabilize or improve appetite, food intake, lean body mass, and body weight for patients with advanced cancer undergoing CT, but rated the level of evidence as low. Studies included in our review that evaluated a sole nutrition intervention of ONS-EPA vs. placebo, an isocaloric diet, or an isocaloric ONS found significantly reduced weight loss and loss of fat free mass, and significantly increased skeletal muscle mass and lean body mass, QoL, and treatment tolerance in the groups receiving ONS-EPA [35,37,39,40]. Because of its potential benefits, ONS-EPA should be considered for cancer patients with weight and lean body mass loss.

Most of the studies included in our review provided an early nutrition intervention, categorized as a nutrition intervention initiated within the first week of cancer treatment or before. The favorable outcomes reported across various studies demonstrate that early nutrition interventions can help improve patients’ prognosis and outcomes. A definitive trial used to document the impact of early vs. late nutrition intervention could be difficult to undertake, since withholding needed nutrition care from patients could be considered unethical. However, a retrospective study or comparison of early nutrition intervention vs. standard care could provide further insights. The only study we identified that specifically examined the timing of nutrition interventions found that the early nutrition intervention group had significantly reduced weight loss, improved treatment tolerance, and fewer CRT breaks (>3 days), CRT delays for toxicity, and unplanned hospitalizations compared to the late nutrition intervention group [28]. Because the poor outcomes observed in the late nutrition intervention group can be detrimental to a patient’s prognosis, early nutrition intervention may improve survival in oncology patients. This is supported by the long-term follow-up study by Ravasco et al., which provided early nutrition interventions and found that the disease-specific survival time in usual diet only (group 3) <ONS with usual diet (group 2) <individualized nutrition counseling (group 1) (*p* < 0.05) [34].

The positive outcomes seen in Meng et al. were also supported in the subset analysis by Kim et al. This analysis evaluated patients based on how far along they were in CT, since around 40% of patients started the study after CT was initiated. The effects of the nutrition intervention were greater in those who received the intervention early at the start of CT. Specifically, body weight, skeletal muscle mass, and fat mass were increased significantly among patients in their first cycle of CT who were receiving ONS, while only fat mass was increased among patients in their second cycle or higher who were receiving ONS [32]. Nonetheless, it is important to note that positive outcomes were also identified when late nutrition interventions were provided. Roca-Rodriguez et al. provided a late nutrition intervention fourteen days after the start of RT and reported a smaller decline in BMI for the intervention group vs. the control group [35]. In the study by Kim et al., providing a late nutrition intervention still resulted in increased fat mass for patients in their second cycle of CT or higher [32].

Our review documented the recent evidence supporting early incorporation of nutrition interventions as a component of cancer therapy for the oncology patient population. In the United States, 90% of cancer care is provided through outpatient cancer centers and clinics [49]. RDNs are uniquely trained to address malnutrition and can provide the individualized nutrition counseling and MNT needed, but they are inadequately staffed in cancer centers. A recent study found that the RDN-to-patient ratio in U.S. oncology centers is 1:2308 [50]. Indeed, only half (53.1%) of oncology centers screen for malnutrition, and a majority (76.8%) of these centers do not bill for nutrition services [50]. The recently introduced US Medical Nutrition Therapy Act of 2020 (H.R. 6971) could help change this and benefit patients by expanding Medicare Part B coverage for MNT for additional medical conditions, including cancer and malnutrition [51].

Individual teams and healthcare providers can also aim to fill existing gaps in malnutrition care and provide early nutrition interventions to improve oncology patients’ outcomes. The recent ESPEN guidelines recommend: “Given the high incidence of nutritional deficits and metabolic derangements among cancer patients, it appears reasonable to monitor relevant parameters regularly in all cancer patients and to initiate interventions early and against all relevant impairments to prevent excessive deficits” [16]. Other professional organizations, such as ASPEN, have not recently published new guidelines on nutrition care for oncology patients. Thus, ESPEN’s recommendation on the implementation of early nutrition interventions and the findings of reviews such as this one can help encourage organizations to consider updating their professional and clinical guidelines to recommend early nutrition interventions. One way to help achieve the recommendations in ESPEN’s guidelines and improve outcomes is through the implementation of a nutrition-focused quality improvement program (QIP). While to date the development and implementation of nutrition-focused QIPs in cancer care appears to be limited [52], several QIPs that included oncology patient populations have illustrated how a nutrition-focused QIP can both improve health and provide economic benefits [53]. Moving forward, nutrition-focused QIPs engaging a multidisciplinary team could be executed in cancer centers to improve nutrition care processes and deliver early malnutrition care.

Our review had several limitations. First, the effects of nutrition interventions in the diverse oncology patient population can be difficult to study and may potentially limit the sample size, number of RCTs, and other research studies performed. Second, our PICOT criteria, electronic search strategy, and databases searched may have excluded studies. For example, our review did not include any studies evaluating enteral or parenteral nutrition as intervention methods. Third, although strict inclusion criteria were applied in our review to minimize the heterogeneity of the studies evaluated, the different study designs and settings, variable nutrition intervention and comparison groups, and the inconsistency of the methods used assess results among the studies likely influenced the findings. Fourth, the accuracy of the results reported from each study cannot be guaranteed, since no original data were accessed. Fifth, we did not complete a formal systematic review or meta-analysis, and thus did not address risk of bias, effect size, or clinical significance. While it has limitations, our review, along with new studies evaluating the benefits of comprehensive nutrition care in patients receiving cancer treatments, could be utilized to help develop nutrition care guidelines, optimize patient-centered care, and subsequently help improve patient outcomes.

## 6. Conclusions

Patients with cancer are at a high risk of malnutrition. This review showed that nutrition interventions in oncology patients receiving active cancer treatment helped improve body weight and BMI, nutrition status, protein and energy intake, QoL, and response to cancer treatments. The reported evidence is limited by the heterogeneity of study designs, small- to medium-sized samples, inconsistent and highly variable nutrition interventions and follow-up periods, and lack of standardized measurements for assessing reported outcomes. Further research is needed to better understand the impact of early nutrition interventions on patients’ outcomes. The optimal duration and timing of nutrition interventions should also be explored. Additionally, future research should investigate the results of implementing a nutrition-focused QIP in cancer centers to improve nutrition care processes and early malnutrition care. This review may help inform the design of quality and comprehensive early nutrition care programs.

## Figures and Tables

**Table 1:** Key search terms to identify studies involving early incorporation of nutrition interventions as a component of cancer therapy.

String	Terms
Cancer	Cancer, neoplasm, tumor, oncology, carcinoma, sarcoma
Treatment	Treatment, chemotherapy, radiation
Nutrition	Nutrition, food, diet
Intervention	Assessment, care plan, plan, planning, counsel, consult, diagnosis, education, evaluation, index, intervention, monitoring, screening, therapy, treatment, oral nutrition supplement (ONS), enteral, parenteral, intravenous, enteric, intragastric, intestinal, intraintestinal, tube, feeding, feeds

**Table 2:** Summary of inclusion and exclusion criteria to identify studies involving early incorporation of nutrition interventions as a component of cancer therapy.

	Inclusion Criteria	Exclusion Criteria
**Population**	Any setting (within last 10 years)	Animal studies
	>18 years of age	<18 years of age
	Diagnosed with cancer	No cancer diagnosis
	Receiving or planning to receive active treatment for cancer diagnosis (unless receiving surgery only)	Not receiving or no plans to receive active treatment for cancer diagnosisOnly receiving surgery as a cancer treatment
	Any nutritional status (well nourished, malnourished, or at-risk of malnutrition)	Pregnant or lactating females
	Studies published within the last 10 years (January 2010 or later)	Studies published before January 2010
**Intervention**	Specified nutrition interventions (singly or in combination) for malnourished patients or those at-risk of malnutrition: - Oral nutritional supplements (ONS) - Enteral nutrition - Parenteral nutrition - Dietary counseling/dietary advice - Formalized nutrition discharge education - ONS coupons and literature on ONS-tailored nutritional care plans at discharge - Nutrition education, post-discharge phone calls - Home visits by registered dietitian nutritionist (RDN)	Nutrition interventions to prevent weight gain Non-commercially available or home-prepared ONS Any of the following (alone or in combination with any other interventions, including the specified nutrition interventions): - Vitamin or mineral supplementation or both - Exercise/physical activity - Behavioral/mental health interventions - Alternative medicine
**Comparison**	Specified nutrition intervention(s) vs. no nutrition intervention(s)	No comparison/control group
	Specified nutrition intervention(s) vs. other specified nutrition intervention(s)	
	Specified nutrition intervention(s) vs. standard of care	
	Early specified nutrition intervention(s) vs. late intervention(s)	
**Duration of Intervention**	>1 week	<1 week
**Outcome**	Anthropometrics - Body weight - Body mass index (BMI)	Outcomes other than the specified health and nutrition outcomes
	Body composition - Muscle mass - Fat mass	
	Nutritional status - Results of malnutrition screening/assesment - Energy intake - Protein intake	
	Functional status - Muscle strength - Handgrip strength - Physical activity	
	Quality of Life (QoL)	
	Hospital readmissions/unplanned hospitalizations	
	Response to treatment - Treatment tolerance - Treatment interruption - Full completion of treatment protocol	
	Emergency Department (ED) visits	
	Complications	
	Morbidity	
	Mortality	
	Healthcare costs	

**Table 3:** Summary of studies included in review of incorporation of nutrition interventions as a component of cancer therapy

Study, Year	Design, Sample Size	Population, Country	Cancer Dx, Cancer Tx	Nutrition Status	Nutrition Intervention(s)	Early or Late Intervention(s), Duration	Outcomes of Nutrition Intervention(s)
Bourdel-Marchasson, 2014 [30]	RCT 341	Older adults (70+ years) France	Lymphoma or carcinoma CT	At risk for malnutrition	Counseling + ONS if needed (intervention group) vs. standard care	Early 3-6 months	↑ Energy intake *ᶲ No difference in weight loss ᶲ No difference in hospitalizations ᶲ No difference in response to cancer treatment ᶲ ↓ Complications (infections) *ᶲ No difference in mortality ᶲ
Cereda, 2018 [31]	RCT 159	Any adults (18+ years) Italy	Head and neck cancer RT or RT plus systemic tx	Any nutrition status	Counseling + ONS (intervention group) vs. counseling only	Early Throughout RT, at 1 month and 3-month follow-up visits after end of RT	↓ Weight loss *ᶲ ↑ Energy intake *ᶲ ↑ Protein intake *ᶲ ↑ Handgrip strength ᶲ ↑ QoL *ᶲ ↑ Treatment tolerance ᶲ
Kim, 2019 [32]	RCT 34	Any adults (20+ years) Korea	Pancreatic and bile duct cancers CT	Patients with a BMI > 30 kg/m^2^ were excluded	Counseling + ONS (intervention group) vs. counseling only	Early for 61.8% of participants (initiated study participation in first cycle of CT) 8 weeks	↑ Nutrition status (measured by PG-SGA) *ᶣ No difference in weight loss ᶲᶣ No difference in skeletal muscle mass ᶲᶣ No difference in FFM ᶲᶣ↑ Fat mass *ᶲᶣ ↑ Energy intake *ᶣ ↑ Protein intake *ᶣ ↑ QoL (fatigue symptoms) ᶣ
Meng, 2019 [28]	Prospective cohort study 78	Adults 18–70 years China	Nasopharyngeal carcinoma CRT	Any nutrition status	Early nutrition intervention (intervention group) vs. late nutrition intervention Intervention for both groups was ONS + EN or PN if needed	Early for participants in the nutrition intervention group; late nutrition intervention group did not receive nutrition support until nutrition-related side effects from treatment developed Nutrition intervention lasted until 3 months after CRT	↓ Weight loss *ᶲ ↓ BMI change *ᶲ ↑ Treatment tolerance (lower incidence of mucositis) *ᶲ ↓ Treatment breaks (>3 days) *ᶲ ↓ Treatment delays for toxicity *ᶲ ↓ Unplanned hospitalizations *ᶲ
Paccagnella, 2010 [29]	Retrospective cohort study 66	Any adults (18+ years) Italy	Head and neck cancer CRT	Any nutrition status	Individualized counseling + ONS/EN if needed (intervention group) vs. standard care	Early Nutrition intervention lasted until 6 months after CRT	↓ Weight loss *ᶲ ↑ Treatment tolerance *ᶲ ↓ Treatment delays *ᶲ ↓ Unplanned hospitalizations *ᶲ
Poulsen, 2014 [33]	RCT 61	Any adults (18+ years) Denmark	GI gynecologic, or esophageal cancer CT and/or RT	Any nutrition status	Counseling + ONS-EPA if desired (intervention group) vs. standard care	Early Between 5–12 weeks, follow-up performed 3 months after treatment	↓ Weight loss *ᶲ ↑ Energy intake *ᶲ ↑ Protein intake *ᶲ No difference in change in FFM ᶲ No difference in change in fat mass ᶲ No difference in QoL ᶲ
Ravasco, 2012 [34]	RCT 111	Any adults (18+ years) Portugal	Colorectal cancer RT followed by surgery + CT	Any nutrition status	Nutrition counseling and education using regular foods (group 1) vs. ONS + usual diet (group 2) vs. usual diet only (group 3)	Early 1.5 months	↑ Nutrition status (measured by PG-SGA; group 1) *ᶲ ↑ BMI (group 1) *ᶲ↑ Energy intake (group 1) *ᶲ ↑ Protein intake (group 1) *ᶲ ↑ Treatment tolerance (measured by late radiotherapy toxicity; group 1) *ᶲ ↑ QoL (group 1) *ᶲ ↓ Mortality (group 1) *ᶲ *Results are from long-term follow-up (range = 4.9–8.2 years) and compared to groups 2 and 3*
Roca-Rodriguez, 2014 [35]	RCT 26	Adults 18–80 years Spain	ENT cancer RT, and CT if needed	Any nutrition status	ONS-EPA (intervention group) vs. isocaloric ONS	Late (14 days after start of RT) 76 days	↓ BMI decline ᶲ
Sanchez-Lara, 2014 [36]	RCT 92	Adults 18–80 years Mexico	Non-small cell lung cancer CT	Any nutrition status	Diet plus ONS-EPA (intervention group) vs. isocaloric diet only Extra calories from ONS were subtracted from intervention group diet so both groups received an isocaloric diet	Early 8+ weeks	↓ Weight loss *ᶲ ↑ LBM *ᶲ ↑ Energy intake *ᶲᶣ ↑ Protein intake *ᶲᶣ ↑ QoL (increased global health status; ᶣ improved fatigue and loss of appetite ᶣᶲ) * ↑ Treatment tolerance (less nausea, vomiting, and neuropathy) *ᶲ No difference in tumor response rate ᶲ No difference in overall survival ᶲ↑ PFS ᶲ
Shirai, 2017 [37]	Retrospective cohort study 179	Adults 18–80 years Japan	GI cancer CT	>5% of pre-illness body weight	ONS-EPA (intervention group) vs. no additional nutritional treatment/placebo	Unknown 6 months	↑ Skeletal muscle mass and LBM *ᶣ No difference in overall survival ᶲ ↑ Treatment tolerance for patients with mGPS of 1 or 2 who received ONS-EPA ᶲ ↑ Prognosis for patients with mGPS of 1 or 2 who received ONS-EPA *ᶲ
Trabal, 2010 [38]	RCT 13	Any adults (18+ years) Spain	Colorectal cancer CT	Excluded patients with severe malnutrition (based on PG-SGA or BMI < 16.5 or >30 kg/m^2^ Patients withdrawn if they developed malnutrition during the study	Counseling + ONS-EPA (intervention group) vs. counseling only	Early 12 weeks	↑ Weight *ᶲ ↑ Energy intake ᶲ ↑ Protein intake ᶲ ↑ QoL (improved fatigue, pain, physical function, social function) ᶲ ↑ Treatment tolerance ᶲ
van der Meij, 2010 [39]	RCT 40	Adults 18–80 years The Netherlands	Non-small cell lung cancer CRT	Any nutrition status	ONS-EPA (intervention group) vs. isocaloric ONS	Early 5 weeks	↓ Weight loss *ᶲ ↓ Loss of FFM *ᶲ No difference in energy intake ᶲ No difference in protein intake ᶲ
van der Meij, 2012 [40]	RCT 40	Adults 18–80 years The Netherlands	Non-small cell lung cancer CRT	Any nutrition status	ONS-EPA (intervention group) vs. isocaloric ONS	Early 5 weeks	↑ QoL (global health status, physical function, cognitive function, social function) *ᶲ ↑ Physical activity (during weeks 3 and 5) *ᶲ No difference in handgrip strength ᶲ ↑ Treatment tolerance (lower incidence of nausea/vomiting) *ᶲ No difference in treatment delays/dose reduction ᶲ No difference in unplanned hospital admissions ᶲ

**Key:** ↑ increased/higher; ↓ decreased/lower; * statistically significant (*p* < 0.05); ᶲ compared to control group/standard of care; ᶣ compared to baselin. **Abbreviations:** BMI, body mass index; CRT, chemoradiotherapy; CT, chemotherapy; Dx, diagnosis; EPA, eicosapentaenoic acid; EN, enteral nutrition; ENT, ear, nose and throat; FFM, fat free mass; GI, gastrointestinal; LBM, lean body mass; mGPS, modified Glasgow Prognostic Score; ONS, oral nutrition supplement; ONS-EPA, oral nutrition supplement containing eicosapentaenoic acid; PFS, progression-free survival; PG-SGA, Patient Generated Subjective Global Assessment; PN, parenteral nutrition; QOL, quality of life; RCT, randomized controlled trial; RT, radiotherapy; Tx, treatment.

## Appendix A

**Table A1:** Full electronic search strategy.

Search Strategy
(Ti,ab((cancer[*1] OR neoplas* OR tumor[*1] OR tumour[*1] OR *carcinoma* OR *sarcoma* OR oncolog*) n/10 (treatment* OR treat* OR chemothera* OR chemo-therap* OR radiat*) n/10 (((diet* OR food OR nutrition*) n/3 (assessment[*1] OR (care n/3 plan) OR plans OR planning OR plan OR counsel* OR council OR diagnos* OR consult* OR (discharg* n/1 education*) OR education* OR evaluation[*1] OR index OR indices OR intervention[*1] OR monitoring[*1] OR “ONS” OR screening[*1] OR therap* OR treatment[*1] OR supplement* OR enteral* OR parental*)) OR “oral nutritional supplement*” OR (parenteral n/1 fluid*) OR ((enteral* OR parenteral* OR intravenous* OR enteric* OR intragastric* OR intestinal* OR intraintestinal* OR tube* OR force*) n/3 (feed OR feeding* OR feeds OR alimentation* OR hyperalimentation*)))) AND ti,ab(((nutrition OR early OR late) n/3 intervention*) OR (standard* n/3 care*)) AND ti,ab,mesh,emb,su,if,au,low,loc,cnt,rg(Australia OR Australian OR Austria OR Austrian OR Belgium OR Belgian OR Bulgaria OR Bulgarian OR Canada OR Canadian OR Croatia OR Croatian OR Cyprus OR Cyprian OR “Czech Republic” OR Czech OR Denmark OR Danish OR Estonia OR Estonian OR “EU-15” OR Finland OR Finnish OR France OR French OR Germany OR German OR Greece OR Greek OR Hungary OR Hungarian OR Iceland OR Icelandic OR Ireland OR Irish OR Italy OR Italian OR Japan OR Japanese OR Latvia OR Latvian OR Lithuania OR Lithuanian OR Luxembourg OR Luxembourgian OR Malta OR Maltese OR Netherlands OR Dutch OR “New Zealand” OR Norway OR Norwegian OR Poland OR Polish OR Portugal OR Portuguese OR Romania OR Romanian OR Slovakia OR Slovakian OR Slovenia OR Slovene OR Spain OR Spanish OR Sweden OR Swedish OR Switzerland OR Swiss OR “United Kingdom” OR British OR “UK” OR “U.K.” OR “United States” OR “US” OR “U.S.” OR “USA” OR “U.S.A.” OR American) AND YR(>=2010) AND la(English)) NOT (dog OR dogs OR cat OR cats OR canine* OR feline* OR porcine* OR pig OR pigs OR piglet* OR cow OR cows OR mice OR mouse OR rat OR rats OR cattle OR veterinar* OR monkey* OR rabbit* OR horse OR horses OR equine* OR zoo OR zoological OR zoology OR zoos OR (animal* n/3 stud*) OR bovine OR geese OR goose OR estuary* OR rodent* OR fish OR fishes OR marine OR dolphin* OR chick OR chicks OR goat OR goats OR ecolog* OR bird* OR sheep* OR zebrafish* OR hamster* OR bat OR bats OR (alternat* n/3 (medicat* OR medicine*)) OR pregnan* OR lactate* OR lactating* OR child* OR adolescen* OR infant* OR infancy OR newborn* OR neonat* OR baby OR babies OR preschool* OR teenage* OR toddler* OR juvenile* OR boy OR boys OR girl* OR pediatric* OR paediatric* OR (“pre” p/0 school*) OR suckling* OR youth OR schoolchild* OR preadolescen* OR ((vitamin* OR mineral*) n/3 supplement*) OR exercis* OR (physical n/3 activ*) OR ((behavior* OR mental*) n/3 health*) OR hospice[*1] OR (palliative n/1 (care OR nursing)))
